# The Possible Clinical Significance of a Decreased Serum Level of Soluble PD-L1 in Discoid Lupus Erythematosus, but Not in Subacute Cutaneous Lupus Erythematosus—A Pilot Study

**DOI:** 10.3390/jcm12175648

**Published:** 2023-08-30

**Authors:** Zsófia Király, Eszter Nagy, Laura Bokor, Anikó Kovács, Márta Marschalkó, Bernadett Hidvégi

**Affiliations:** 1 Department of Dermatology, Venereology and Dermatooncology, Semmelweis University, 1085 Budapest, Hungarymarschalko.marta@med.semmelweis-univ.hu (M.M.); hidvegi.bernadett@med.semmelweis-univ.hu (B.H.); 2 Department of Laboratory Medicine, Semmelweis University, 1094 Budapest, Hungary; nagy.eszter@orfi.hu; 3 National Institute of Locomotor Diseases and Disabilities, 1122 Budapest, Hungary

**Keywords:** cutaneous lupus erythematosus, discoid lupus erythematosus, subacute cutaneous lupus erythematosus, soluble PD-1, soluble PD-L1

## Abstract

Cutaneous lupus erythematosus (CLE) is an autoimmune skin disease with various clinical forms, including the subtypes of discoid lupus erythematosus (DLE) and subacute cutaneous lupus erythematosus (SCLE). The altered function of the programmed cell death 1/programmed cell death ligand 1 (PD-1/PD-L1) axis in CLE pathogenesis has been suggested. Here, the soluble forms of PD-1 (sPD-1) and PD-L1 (sPD-L1) were explored in untreated DLE and SCLE. Levels of sPD-1 and sPD-L1 were determined by enzyme-linked immunosorbent assay in serums of 21 DLE, 18 SCLE, 13 systemic lupus erythematosus (SLE) patients and 20 healthy controls (HCs). Differences between patient groups and HCs, and the association between clinical activity of skin symptoms and sPD-1/sPD-L1 levels were analyzed with Mann–Whitney U-test and Spearmann’s correlation. Regarding sPD-1 levels, no statistically significant differences were found between DLE and SCLE groups, nor compared to HCs. As for sPD-L1, a significantly lower level was found in the DLE group compared to the SCLE and HC groups (*p* = 0.027 and *p* = 0.009, respectively). In SLE, significantly higher sPD-1 was found compared to HCs (*p* = 0.002). No association between skin symptom activity and sPD-1/sPD-L1 levels was found in CLE. Alterations of the inhibitory effect of sPD-L1 on T-cell activity might elucidate the differences between DLE and SCLE.

## 1. Introduction

Cutaneous lupus erythematosus (CLE) is an autoimmune skin disease characterized by a wide range of clinical manifestations and a pathomechanism that is still not completely understood. The pathogenesis of CLE is multifactorial; it is likely that certain environmental factors (such as UV light or medications) may induce the increased cell death of keratinocytes in the skin. Additionally, clearance of these cells is probably also impaired, leading to the accumulation of nuclear substances in the tissues. Subsequently, these factors can activate both the innate and the adaptive immune system, triggering the release of cytokines, chemokines and interferons, and thus, a self-reinforcing cycle develops [1].

According to the Düsseldorf classification, the subtypes of CLE are acute cutaneous lupus erythematosus, subacute cutaneous lupus erythematosus (SCLE), chronic cutaneous lupus erythematosus (CCLE) and intermittent cutaneous lupus erythematosus [2]. CCLE is further categorized into subtypes such as discoid lupus erythematosus (DLE), lupus erythematosus panniculitis and Chilblain lupus. Among these subtypes, DLE is the most common form of CCLE. Clinical features of DLE are discoid, livid-erythematous plaques with deep infiltration and a tendency to scar. These plaques typically present on the face, scalp and neck (localized DLE), or on the trunk and extremities as well (generalized DLE), while association with autoantibodies is rare [3,4,5]. Another quite common form of CLE is SCLE, which is characterized by erythematous scaling annular or papulosquamous eruptions on sun-exposed areas of the body. These lesions show no tendency for scarring and an association with autoantibodies (mainly directed against Sjögren’s syndrome-related antigen A (SS-A/Ro)) is more frequent [3,4]. Not only is the pathogenesis of lupus erythematosus unclear, the background of the various clinical manifestations of the wide range of the lupus spectrum is also obscure. Recent research has shown that there are variations in the infiltrate composition within clinical subtypes of CLE [6]. A study by de Vos et al. revealed predominantly interferon-driven expression markers in SCLE, whereas DLE samples exhibited a higher presence of B-cell infiltration [6]. The investigation into the role of these “skin-specific” B-cells in CLE is currently ongoing; however, their distinct distribution in the skin might be a promising lead towards uncovering further differences in the pathogenesis.

Recently, the role of immune checkpoint molecules has gained theoretical and practical significance in various fields of medicine, including autoimmunity [7,8]. The programmed cell death 1 (PD-1) receptor is a cell surface molecule on activated T-cells, while its ligand, the programmed cell death ligand 1 (PD-L1) is expressed by various cells of the body (e.g., lymphocytes and keratinocytes). It is generally acknowledged that soluble variations of these proteins are also present, and they are likely to influence the PD-1/PD-L1 axis; however, their roles are currently under investigation [7,9].

The connection between PD-1 and PD-L1 plays a crucial part in maintaining peripheral tolerance. Thus, soluble PD-1 (sPD-1) and soluble PD-L1 (sPD-L1) could potentially influence the PD-1/PD-L1 axis and might lead to the induction of autoimmunity. The clinical roles of sPD-1 and sPD-L1 as biomarkers and/or therapeutic targets are nowadays widely investigated in cancer research, while an emerging number of studies explore the possible roles of these proteins in the development and course of autoimmune diseases like SLE or rheumatoid arthritis [10,11,12,13].

It is well known that amongst many other drugs, programmed cell death 1 (PD-1) or programmed cell death ligand 1 (PD-L1) checkpoint inhibitors might induce SCLE, but not DLE, and thus, the PD-1/PD-L1 pathway may play a different role in the pathogenesis of different forms of CLE [14,15,16,17]. It is plausible that checkpoint inhibitors could activate pathological pathways associated with T-cell activity, leading to the development of SCLE. Given that a more notable B-cell infiltration is present in DLE [6], the occurrence of DLE induction in response to checkpoint inhibitors would be unlikely. In our previous pilot study focusing on this subject, we discovered resemblances between checkpoint-inhibitor-induced SCLE and idiopathic SCLE in terms of PD-1 and PD-L1 expression within the skin. Notably, we observed a markedly lower expression of PD-L1 in keratinocytes within DLE samples compared to the SCLE samples [18]. This interesting finding prompted us to explore this topic further in another experimental setting.

To date, there are no data regarding sPD-1 and sPD-L1 in CLE; however, they might influence the pathogenesis and course of CLE. In this study, we aimed to explore the possible differences between subgroups of CLE (DLE and SCLE) regarding sPD-1 and sPD-L1. Another objective was to investigate the roles of sPD-1 and sPD-L1 in CLE and their connection to the clinical activity of skin symptoms.

## 2. Materials and Methods

### 2.1. Patients and Sample Collection

Patients were diagnosed with DLE, SCLE and SLE at the Department of Dermatology, Venereology and Dermatooncology of Semmelweis University. No concomitant SLE or other systemic autoimmune disease was observed in any of the DLE or SCLE patients, while all SLE patients fulfilled the 2019 EULAR/ACR criteria. The diagnosis of CLE was based on clinical characteristics and histological findings. Further categorization into DLE subgroup was based on head–neck distribution of scarring plaques and lack of autoantibodies, while further diagnosis of SCLE was supported by the typical annular or psoriasiform lesions in sun-exposed areas and the presence of autoantibodies, mainly anti-SS-A/Ro antibodies. In this setting, the group of SLE patients served as a positive control group, since some data regarding sPD-1 and sPD-L1 levels in this disease are available. Severity of the lupus skin lesions was evaluated using the activity domain of cutaneous lupus disease area and severity index (CLASI-A) at the time of serum sample collection [19]. The Systemic Lupus Erythematosus Disease Activity Index 2000 (SLEDAI-2K) was employed to evaluate disease activity in SLE patients [20].

Serum samples from 21 DLE, 18 SCLE and 13 SLE patients were collected before administration of any topical and systemic medication for lupus erythematosus. Samples from 20 healthy age- and sex-matched subjects were used as healthy controls (HCs). All samples were stored at −80 °C before use.

### 2.2. Measurement of Autoantibodies

Serum samples of CLE and SLE patients were examined for the presence of antinuclear antibodies (ANA), antibodies to double-stranded DNA (dsDNA), SS-A/Ro, Sjögren’s syndrome-related antigen B (SS-B/La), phospholipids (Cardiolipin (CL) and β2-glycoprotein I (β2-GPI)). ANA were assessed with indirect immunofluorescence on a HEp-2 substrate with a commercially available kit (NOVA Lite HEp-2 IgG, Inova Diagnostics, San Diego, CA, USA). Antibodies to double-stranded DNA (dsDNA), CL (IgG and IgM) and β2-GPI (IgG and IgM) were quantified with a chemiluminescent immunoassay by using commercially available kits (QUANTA Flash dsDNA, QUANTA Flash aCL IgG, QUANTA Flash aCL IgM, QUANTA Flash β2GP1 IgG, QUANTA Flash β2GP1 IgM, Inova Diagnostics, San Diego, CA, USA), while anti-SS-A/Ro and anti-SS-B/La antibodies were assayed with commercially available ELISA kits (QUANTA Lite SS-A, QUANTA Lite SS-B, Inova, San Diego, CA, USA). Testing for autoantibodies was performed according to the manufacturer’s instructions.

### 2.3. Measurement of Serum sPD-1 and sPD-L1 Levels

Serum levels of sPD-1 and sPD-L1 were measured using commercially available human PD-1 and human/cynomolgus monkey PD-L1/B7-H1 ELISA kits (Quantikine, R&D Systems, Minneapolis, MN, USA) according to the manufacturer’s instructions. Standards (consisting of a seven-point serial dilution) and samples were added to 96-well plates precoated with a monoclonal antibody specific for human PD1 and human B7-H1, respectively. A three-level control set for both human PD1 and human PD-L1 (Quantikine, R&D Systems, Minneapolis, MN, USA) was performed in parallel with the samples during each run. The specific binding protein was detected with an enzyme-linked monoclonal anti-PD1 antibody for sPD-1 and a polyclonal anti-PD-L1 antibody for sPD-L1 and then revealed with TMB substrate. The reaction was stopped by adding 2 N H_2_SO_4_ and the plates were measured at 450 nm by the microplate reader (Multiskan^®^ EX, Thermo Fisher Scientific, Waltham, MA, USA). The concentration of PD-1 and PD-L1 (pg/mL) was calculated using the 4-point-fit calibration curve of standard dilutions. The detection range was 15.6–1000 pg/mL for sPD-1, with a minimum detectable dose of 3.27 pg/mL and 25.0–1600 pg/mL for sPD-L1, with a minimum detectable dose of 4.52 pg/mL.

### 2.4. Statistical Analysis

The Shapiro–Wilk test of normality was used to evaluate the normality of serum levels of sPD-1 and sPD-L1 and indicated non-normal distributions of our data. Hence, non-parametric Mann–Whitney U-test was used for two-group analysis. Correlations between sPD-1, sPD-L1 and CLASI were evaluated by using Spearmann’s rank correlation coefficient. *p* values lower than 0.05 were considered indicators of a significant difference.

## 3. Results

### 3.1. Characteristics of the Study Population

**Figure 1:**
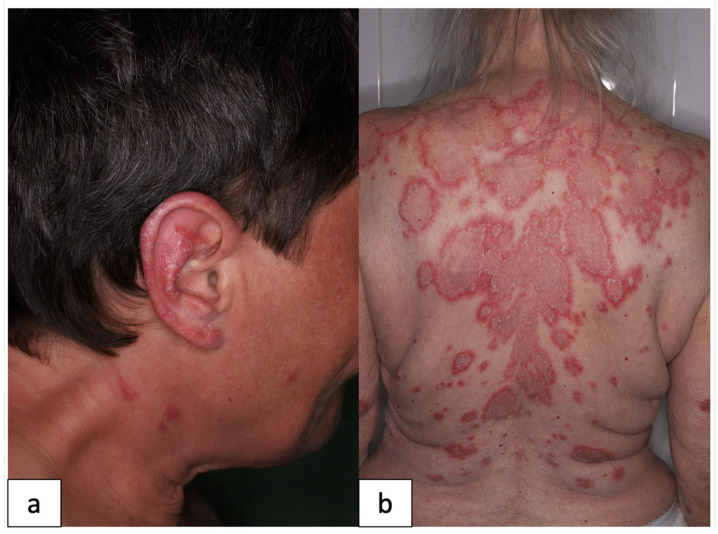
Representative clinical pictures of our patients. DLE (**a**) presents as scaling, livid-erythematous plaque on the ear. In SCLE (**b**), annular, erythematous plaques are shown on the back. (DLE: discoid lupus erythematosus, SCLE: subacute cutaneous lupus erythematosus).

All patients in the DLE group presented with localized DLE, and none of them had generalized skin symptoms. Among the 18 SCLE patients, 13 demonstrated annular symptoms, while the remaining 5 displayed psoriasiform lesions. The median CLASI-A score was 6 for all three patient groups, including DLE, SCLE and SLE groups. Representative pictures of our DLE and SCLE patients are shown on Figure 1. Notably, all SLE patients exhibited skin symptoms (3 ACLE, 5 SCLE, 3 DLE and 2 other CLE). Some CLE patients displayed mild organ manifestations; two SCLE patents had arthritis, one SCLE patient had leukopenia and one other proteinuria. Furthermore, in addition to their skin lesions, our SLE patients presented with various organ manifestations, eight cases of arthritis, three of proteinuria, five of leukopenia and one of thrombocytopenia. Hypocomplementemia was observed in eight SLE cases. The median Systemic Lupus Erythematosus Disease Activity Index 2000 (SLEDAI-2K) score of the SLE group was 8. Regarding their antibody profiles, ANA was positive in 4 of 21 DLE and 12 of 18 SCLE patients (dilution 1:160). Anti-SS-A/Ro antibodies were present in three DLE patients, eleven SCLE patients and seven SLE patients, while anti-SS-B/La antibodies were detected in one DLE patient, ten SCLE patients and five SLE patients. Among SLE patients, 9 of 13 tested positive for anti-dsDNA antibody, whereas none of the DLE or SCLE patients showed positivity. Additionally, two DLE, one SCLE and two SLE patients had anti-CL, while one DLE, one SCLE and four SLE patients had anti-β2-GPI antibodies. Characteristics of the patient groups are shown in Table 1.

### 3.2. Comparison of Serum sPD-1 Levels in Patient Groups and Healthy Controls

**Figure 2:**
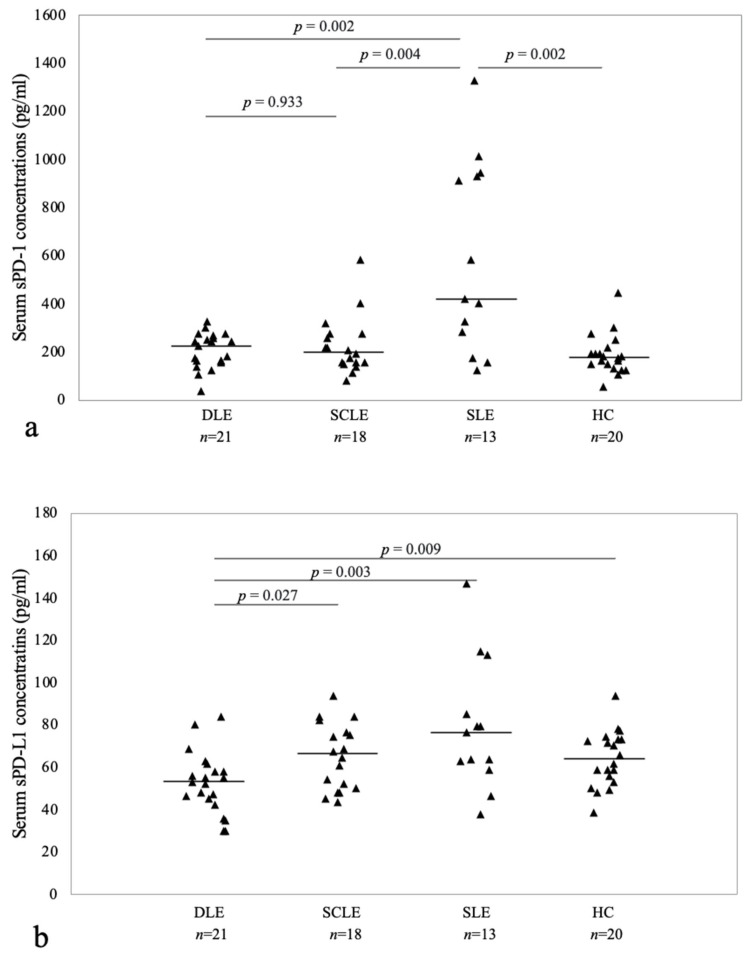
Serum sPD-1 and PD-L1 levels in DLE, SCLE, SLE and HCs. Serum levels of sPD-1 (**a**) were significantly higher among SLE patients than in HCs (*p* = 0.002); however, no significant difference between DLE, SCLE and HC groups was observed. Serum levels of sPD-L1 (**b**) were significantly decreased in the DLE group compared to the HCs (*p* = 0.009), while no significant differences between SCLE patients and HCs were observed. (sPD-1: Soluble programmed cell death 1, sPD-L1: soluble programmed cell death ligand 1, DLE: discoid lupus erythematosus, SCLE: subacute cutaneous lupus erythematosus, SLE: systemic lupus erythematosus, HCs: healthy controls).

The median serum level of sPD-1 in DLE, SCLE, SLE and HC groups were 225.35 pg/mL and 200.97 pg/mL, 420.12 pg/mL and 177.01 pg/mL, respectively. Notably, the serum levels of sPD-1 were significantly higher among SLE patients than in HCs (*p* = 0.002). No statistically significant differences were observed between the DLE and SCLE groups (*p* = 0.933) when compared to each other or to the HC group. However, both DLE and SCLE groups exhibited significant differences in sPD-1 when compared to the SLE (*p* = 0.002 and *p* = 0.004, respectively) (Figure 2a).

### 3.3. Comparison of Serum sPD-L1 Levels in Patient Groups and Healthy Controls

Concerning the serum levels of sPD-L1, the median values observed in DLE, SCLE, SLE and HC groups were 53.52 pg/mL, 66.4 pg/mL, 76.55 pg/mL and 64.1 pg/mL, respectively. Notably, the DLE group exhibited a significant decrease in sPD-L1 serum levels compared to the HC group (*p* = 0.009). However, no significant differences were observed between the SCLE and SLE groups when compared to the HC group. Furthermore, a significantly lower sPD-L1 level was found in the DLE group compared to both the SCLE and SLE groups (*p* = 0.027 and *p* = 0.003, respectively) (Figure 2b).

### 3.4. Correlations between Serum sPD-1, sPD-L1 and the Activity of Skin Symptoms

The medians of the CLASI-A scores of DLE and SCLE patients (*p* = 0.18) did not show any significant differences. No significant correlation between serum levels of sPD-1, sPD-L1 and CLASI-A scores in DLE and SCLE groups were observed.

## 4. Discussion

It is generally accepted that dysregulation of the PD-1/PD-L1 pathway is implicated in the pathogenesis of various types of cancers, infections and autoimmune diseases [7,10]. sPD-1 is generated through alternative splicing and seems to bind to PD-L1, thereby interfering with the binding of membrane-bound PD-1. This interaction subsequently leads to the activation of T-cell functions. sPD-L1 can also arise from alternative splicing or cleavage of the membrane-bound form by matrix-metalloproteases. The function of sPD-L1 is currently a subject of ongoing discussion, although it is most likely to exhibit similar inhibitory effects on T-cell activity as the membrane-bound form does [7].

The roles of sPD-1 and sPD-L1 as prognostic factors are thoroughly investigated in the field of oncology. Elevated levels of sPD-1 have been found in many types of tumorous diseases like non-small cell lung cancer (NSCLC), hepatocellular carcinoma, diffuse large B-cell lymphoma or metastatic melanoma [9,21]. The pretherapeutic higher plasma level of sPD-1 was found to be associated with disease severity, prognosis and clinicopathological features; however, these findings are sometimes inconsistent [9]. A recent meta-analysis found that elevated levels of sPD-L1 were significantly associated with worse overall survival in patients treated with an immune checkpoint inhibitor; however, this varies between different tumor types. This association was strong in NSCLC but weaker in melanoma, renal cell and esophageal cancer [8].

Several publications on the PD-1/PD-L1 axis in non-oncologic pathologies are currently available, although the mechanism of this largely complex pathway in autoimmunity is not totally clear yet [7]. As mentioned before, sPD-1 enhances T-cell activity, while the effect of sPD-L1 on T-cell function is currently debated; however, it most probably has an inhibitory effect on T-cells, similar to its membrane-bound form [22]. In SLE, one study observed increased sPD-1 levels among patients compared to healthy controls, while in another study, an elevated level of sPD-1 was found only among SLE patients with high disease activity in comparison to those with low disease activity and healthy controls [11,12]. The observed elevated serum sPD-1 level in our SLE group aligns with these findings, and the median values we obtained are comparable to the measurements reported by Hirahara et al. in individuals with high-disease-activity SLE (420.12 and 482.7 pg/mL, respectively). Concerning sPD-L1 levels, Du et al. found a significant increase in SLE patients in contrast to the observations of Hirahara et al., who found no such difference [11,12]. In our study, no statistically significant difference was detected in sPD-L1 levels between SLE and HC groups. The inconsistent findings regarding sPD-1 and sPD-L1 levels in immune-mediated diseases might be partially attributed to factors such as the type and activity of the disease, as well as variations in the detection ranges and sensitivities of the ELISA kits used for measurement [7]. With regard to psoriasis, prior research investigating serum sPD-1 levels in psoriatic patients found no significant difference compared to HCs [23]. Another study investigating immune thrombocytopenia (ITP) found elevated sPD-1 levels in both newly diagnosed and chronic ITP, while sPD-L1 was found to be lower only in the newly diagnosed group when compared to healthy controls [24]. In a study conducted by Yanaba et al., elevated levels of sPD-1 were observed in patients with diffuse cutaneous systemic sclerosis, but not in those with limited cutaneous systemic sclerosis. Conversely, sPD-L1 was found to be elevated in both groups compared to healthy controls. In their study, serum sPD-1, but not sPD-L1, was positively correlated with the severity of skin sclerosis [25]. As part of our study, the severity of lupus skin lesions was evaluated with the CLASI-A score; however, no relationship between serum levels of sPD-1 or sPD-L1 and activity of skin symptoms was observed.

To the best of our knowledge, this is the first study investigating the roles of sPD-1 and sPD-L1 in the pathogenesis of CLE. Recent reports of PD-1/PD-L1 inhibitors triggering SCLE but not DLE raised the possibility of potential variances within the PD-1/PD-L1 axis between these two distinct clinical subtypes [14,15,16,17]. In the present study, the serum levels of sPD-1 and sPD-L1 were measured in untreated DLE and SCLE patients. These measurements were then compared between the two patient groups as well as with SLE patients and a control group of healthy individuals. Furthermore, the potential relationship between the levels of these proteins and the activity of skin symptoms was investigated. No significant differences of sPD-1 serum levels were found between DLE, SCLE and HC groups; however, a significant decrease in sPD-L1 serum levels was observed in the DLE group compared to both the HC and SCLE groups.

Based on our above-mentioned results, it may be concluded that the increased level of sPD-1 might contribute to the development of systemic autoimmune diseases, while it has smaller effect in the pathogenesis of diseases that affect mainly the skin. In another experimental setting, our team found markedly lower keratinocyte PD-L1 expression in DLE compared to SCLE (the normal skin was negative), providing additional insights into variations in the PD-1/PD-L1 axis between the clinical subtypes of CLE [18]. Based on our findings and former publications, it is possible that the insufficiency of inhibitory effect of sPD-L1 on T-cell activity might contribute to the differences between the clinical presentations of DLE and SCLE.

The complex mechanism of the PD-1/PD-L1 axis is intertwined with multiple cellular signaling pathways and diverse cell types, making it challenging to investigate isolated parts of this system. An important question for future research would be whether sPD-1 and sPD-L1 originate from keratinocytes, lymphocytes or other cells.

Future studies might help clarify the role of sPD-1 and sPD-L1 in the development and course of CLE. Understanding the PD-1/PD-L1 axis in forms of CLE may lead us to better understand the key differences in the pathogenesis and prognosis of DLE and SCLE, and why SCLE is triggered by checkpoint inhibitors.

## Figures and Tables

**Table 1:** Characteristics of the patient groups.

	DLE	SCLE	SLE
Patient demographics			
Female/male ratio	19/2	13/5	9/4
Age (mean ± SD)	47.6 ± 14.5	62.9 ± 15	49 ± 17.9
Autoantibody positivity			
ANA	4/21	12/18	13/13
anti-SS-A/Ro	3/21	11/18	7/13
anti-SS-B/La	1/21	10/18	5/13
anti-dsDNA	0/21	0/18	9/13
anti-CL	2/21	1/18	2/13
anti-β2-GPI	1/21	1/18	4/13
Organ manifestations			
Rash	21/21	18/18	13/13
Oral ulcer	0/21	0/18	2/13
Alopecia	13/21	4/18	2/13
Arthritis	0/21	2/18	8/13
Proteinuria	0/21	1/18	3/13
Leukopenia	0/21	1/18	5/13
Thrombocytopenia	0/21	0/18	1/13
Hypocomplementemia	0/21	0/18	8/13
CLASI-A score (median, min-max)	6 (1–14)	6 (1–18)	6 (3–21)
SLEDAI-2K score (median, min-max)	NA	NA	8 (2–20)

(DLE: discoid lupus erythematosus; SCLE: subacute cutaneous lupus erythematosus; SLE: systemic lupus erythematosus; ANA: antinuclear antibodies; anti-SS-A: anti Sjögren’s syndrome-related antigen A; anti-SS-B: anti Sjögren’s syndrome-related antigen B; anti-dsDNA: anti-double-stranded DNA; anti-CL: anti-Cardiolipin; anti-β2-GPI: anti-β2-glycoprotein I; NA: not applicable; CLASI-A: activity domain of cutaneous lupus disease area and severity index; SLEDAI-2K: Systemic Lupus Erythematosus Disease Activity Index 2000).

## Data Availability

No new data were created or analyzed in this study. Data sharing is not applicable to this article.
